# Visuospatial memory deficit, plasma p-tau217, and Aβ42/Aβ40 ratio enhance sensitivity to identify Aβ PET positivity in individuals with SCD

**DOI:** 10.1016/j.tjpad.2026.100525

**Published:** 2026-02-27

**Authors:** Qinjie Li, Lin Huang, Ying Wang, Yihui Guan, Fang Xie, Qihao Guo

**Affiliations:** a Department of Gerontology, Shanghai Jiao Tong University Affiliated Sixth People’s Hospital, Shanghai, 200233, China; b Department of nuclear medicine & PET Center, Huashan Hospital, Fudan University, Shanghai, China

**Keywords:** Alzheimer's disease, Subjective cognitive decline, Visuospatial memory, p-tau217, Amyloid β, PET

## Abstract

**Introduction:**

We hypothesize that specific cognitive assessments and plasma biomarkers may exhibit heightened sensitivity during the stage of subjective cognitive decline (SCD). The integration of these plasma biomarkers and cognitive assessments could enhance the ability to predict beta-amyloid (Aβ) pathology in individuals with SCD.

**Methods:**

A total of 231 participants, including 74 normal controls (NC) and 157 SCD, underwent Aβ and tau PET scans and blood testing for Aβ40, Aβ42, p-tau181, p-tau217, NfL, and GFAP. Cognitive assessments, plasma biomarkers, tau PET SUVr, and demographics were compared between Aβ+ and Aβ− groups within NC and SCD. The least absolute shrinkage and selection operator (LASSO) and logistic regression were employed to perform variable selection and develop predictive models.

**Results:**

We observed significantly worse global cognition, visuospatial memory performance, executive function, and metamemory, as well as higher tau PET SUVr, elevated levels of p-tau217, p-tau181, and GFAP, and lower Aβ42/Aβ40 ratios in SCD Aβ+ compared to SCD Aβ-. The model incorporating BVMT-LD and p-tau217 achieved a slightly higher AUC than the model using p-tau217 and Aβ42/Aβ40 (0.94 vs. 0.93). Partial correlation analyses indicated that both auditory verbal memory (AVLT-LD) and visuospatial memory (BVMT-LD) were significantly negatively associated with p-tau217, whereas only AVLT-LD demonstrated a significant negative association with tau pathology severity.

**Conclusion:**

Visuospatial memory deficit and plasma p-tau217 are powerful biomarkers for identifying Aβ+ in SCD. Auditory verbal memory links to tau pathology severity, while visuospatial memory is more sensitive to Aβ deposition, supporting early intervention to prevent AD progression.

## 1 Introduction

Alzheimer’s disease (AD) is the most common cause of dementia, imposing heavy socioeconomic and caregiving burdens globally [1,2]. In China, over 9.8 million adults aged ≥60 years have dementia, with 38.8 million affected by mild cognitive impairment (MCI) [3]. According to the international consensus on the biological definition, AD is recognized as a continuum defined by underlying pathological processes [2]. Aβ deposition in the brain is widely recognized as AD’s core pathogenic mechanism. Current innovative treatments—particularly Aβ-targeting immunotherapies and disease modifying therapies (DMTs) in clinical trials—are more effective for MCI and early-stage AD patients [4,5]. However, the early stage of AD is frequently underdiagnosed, and is often accompanied by low rates of follow-up and treatment adherence [6].

Under the current consensus, the diagnosis of AD involves a comprehensive neuropathological framework, including amyloid-β deposition (A), pathologic tau (T), and neurodegeneration (N) [7]. Aβ positron emission tomography (PET) imaging enables the in vivo detection of fibrillar amyloid-β deposits within neuritic plaques. However, the high cost and limited accessibility of amyloid PET imaging for screening purposes have limited its broader clinical application. The blood-based biomarkers offer advantages in terms of being less invasive, more cost-effective and better feasibility. Previous studies have confirmed that blood-based biomarkers, including Aβ42, Aβ40, p-tau181, p-tau217, and neurofilament light chain (NfL), show significant correlations with cerebrospinal fluid (CSF) levels and the standard uptake value ratio (SUVR) obtained from amyloid PET imaging [[8], [9], [10]]. Additionally, previous studies have reported elevated levels of plasma p-tau181, p-tau217, glial fibrillary acidic protein (GFAP) and NfL [8,[11], [12], [13], [14]], and decreased levels of Aβ42/Aβ40 ratio [8,11,12] in individuals with amyloid positive compared to those with amyloid negative within the same cognitive status.

SCD criteria are defined by two major features under the research background, (1) a self-experienced persistent decline in cognitive capacity, compared with a previously normal cognitive status, which is unrelated to an acute event, and (2) normal performance on standardized cognitive tests used to classify MCI [15]; meanwhile, the cognitive decline is not associated with any physical or mental conditions, nor with the use of medication, alcohol, or other substances [16]. The estimated prevalence of Alzheimer’s disease neuropathological changes (ADNCs) increases with age: from <8 % in individuals aged 58–69.9 years to 65.2 % in those aged >90 years [17]. Among adults aged ≥70 years, ADNC prevalence varies by cognitive status: 23.5 % in the cognitively unimpaired group, 32.6 % in those with mild cognitive impairment (MCI), and a notably higher 60 % in people with AD dementia [17]. One meta-analysis reported that the annual conversion rate from SCD to MCI was 6.6 % [18], and the conversion rate to dementia was 2.3 % [18]; in comparison, the annual conversion rate from normal cognition (without SCD) to MCI was only 1 % [18]. Patients with SCD may experience mild emotional fluctuations, such as anxiety, depression, or social withdrawal [[19], [20], [21], [22], [23]]; however, these symptoms are generally not severe and do not significantly interfere with their daily function [19]. At the SCD stage, individuals may exhibit a subtle but objectively measurable cognitive decline relative to cognitively unimpaired older adults, particularly in memory domains such as episodic and semantic memory [24]. Given that significant neuronal loss and irreversible cognitive impairments are already present in individuals with MCI in Alzheimer’s continuum, early detection prior to the MCI stage may provide a more favorable window for intervention [25].

Most current screening tools are designed to detect clinical cognitive impairment, rather than the pre-MCI stage. For example, the Petersen/Winblad[26] and the Jak/Bondi criteria[27]defined cognitive impairment with 1.5 or 1 standard deviation (SD) below normative expectations. Additional tools, including Addenbrooke’s Cognitive Examination-III (ACE-III) [28] and Montreal Cognitive Assessment Basic Version (MoCA-B) [29], use cutoff values validated in large samples yet are insensitive to the pre-AD stage. Innovative cognitive metrics are essential to facilitate the detection of prodromal AD stages, which is critical for research and clinical trials [30]. Just as episodic memory impairment is most commonly observed in MCI due to AD (prodromal AD) who later progress to AD dementia [26,31], we hypothesize that certain cognitive functions may also be particularly vulnerable during the SCD stage.

In this study, we investigated subtle cognitive alterations between Aβ- and Aβ+ in individuals with SCD and cognitively healthy controls across a broad range of cognitive domains, including memory, executive function, language, visuo-spatial abilities, attention, metacognition, and mood. We aim to identify certain differences that may serve as potential cognitive markers during the SCD stage. Subsequently, we evaluated the predictive performance of combining accessible cognitive assessments with non-invasive biomarkers for identifying amyloid pathology in individuals with SCD. In addition, we examined the correlation between cognitive assessments and tau pathology severity to identify potential cognitive markers that may be associated with the severity of tau pathology in the SCD stage.

## 2 Methods

### 2.1 Participants

**Fig. 1:**
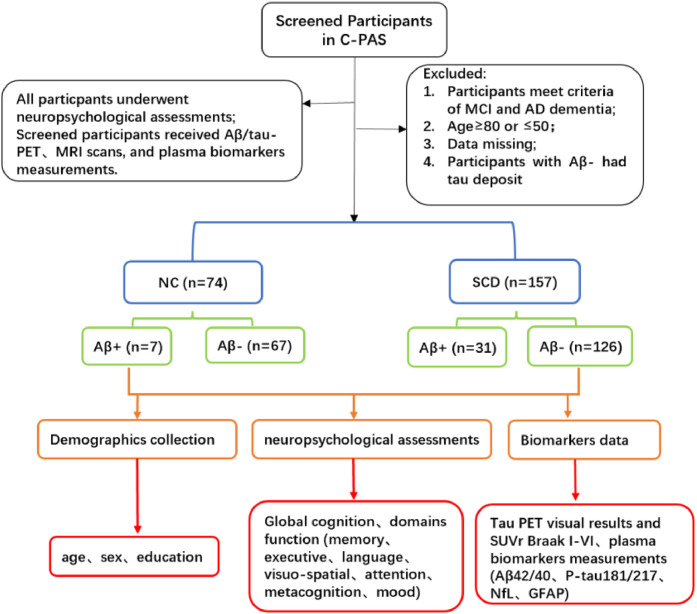
A flow chart of participants screened.

A total of 231 participants (57.14 % females; mean age = 66.45 ± 7.59 years; age range: 50–80 years) were enrolled from the Chinese Preclinical Alzheimer’s Disease Study (C-PAS) from China between 2019 and 2025 [32]. C-PAS is a long-term, ongoing cohort study on the Alzheimer’s disease spectrum in China, with a particular focus on its preclinical stages [32], with recruitment sites including the Department of Gerontology at Shanghai Jiao Tong University Affiliated Sixth People’s Hospital and the PET Center at Huashan Hospital, Fudan University. The C-PAS cohort was designed to match the demographic characteristics of the elderly population in eastern Chinese areas. Eligible participants were aged 50–80 years, with normal or corrected-to-normal visual and auditory function to ensure the completion of neuropsychological assessments. Exclusion criteria included a history of head trauma, alcohol or substance abuse, other neuropsychiatric disorders (e.g., schizophrenia, Parkinson’s disease), and a diagnosis of mild cognitive impairment (MCI) or dementia. All participants completed a standardized assessment battery, including comprehensive neuropsychological tests, Aβ/tau PET scans, 3.0 T brain MRI, and plasma biomarker measurements. Fig.1 illustrated the inclusion and exclusion criteria. This study was approved by the ethics committee of the Shanghai Sixth People’s Hospital. All participants signed an informed consent.

### 2.2 Neuropsychology and diagnostic criteria

All participants complete a comprehensive battery of neuropsychological measures, including Chinese version of Addenbrooke’s Cognitive Examination-III (ACE-III) [28], Montreal Cognitive Assessment Basic Version (MoCA-B) [29] and domain function, including memory, executive, language function, visuo-spatial function, attention, metacognition, and mood. The memory domain assessment includes the auditory verbal learning test-Huashan (AVLT-H) [33] and the Chinese version of brief visuospatial memory test (BVMT) revised by the team of professor Qihao Guo [34,35]. AVLT-LD and AVLT-R denote the long-term delayed recall and recognition components of the AVLT, respectively. BVMT-LD and BVMT-R denote the long-term delayed recall and recognition components of the BVMT, respectively. The BVMT-L denotes the difference between the third and first recall trials, reflecting the individual's learning capacity. The BVMT-F denotes the difference between the sixth and third recall trials, which reflects the individual's degree of forgetting. The executive domain includes the shape trails test parts A (STT-A), and trails test parts B (STT-B) [36]. The language domain assessment incorporates the Boston Naming Test (BNT) and the animal verbal fluency test (AFT) [37,38]. The visuo-spatial domain includes the Judgment of Line Orientation (JLO) [39]. The attention domain test employs the digit span test (DST), which comprises two components: the digit span forwards (DSTf) and the digit span backwards (DSTb) [40]. The metacognition assessment includes the degree of confidence (DOC), which comprises short-term delayed recall and long-term delayed recall (DOC-SD and DOC-LD) [41,42], as well as self-report memory questionnaire (SMQ). The mood assessment includes the Hamilton depression rating scale (HAMD) and the Hamilton anxiety rating scale (HAMA) [43,44].

NC individuals were defined as having no subjective cognitive complaints (verified by informants), normal performance on all standardized cognitive tests (without any subtle impairment), and no evidence of neurodegeneration or amyloid deposition [45]. The criteria for SCD were firstly according to the following Jessen’s criteria [15]: (1) a self-experienced persistent decline in cognition (decline in memory domain must be included), which is unrelated to an acute event; (2) normal performance on standardized cognitive tests used to classify mild cognitive impairment, adjusted for age, sex, and education; secondly, met more than three features of SCD plus [19], such as, age at onset of subjective cognitive decline ≥ 60 years; onset of subjective cognitive decline within the last 5 years; feeling of worse performance than others of the same age group; concerns (worries) associated with subjective cognitive decline; a confirmed cognitive decline by the informants. The MCI criteria used in this cohort were based on the Jak/Bondi actuarial neuropsychological criteria [27], (1) impaired scores, defined as >1 SD below the normative mean, on two of the six neuropsychological indexes in the same cognitive domain; (2) impaired scores (>1 SD) in each of the three cognitive domains; (3) Functional Assessment Questionnaire (FAQ) score≥9. MCI criteria were referenced only for exclusion purposes to ensure no MCI participants were included in the study; all enrolled participants (NC and SCD) did not meet MCI diagnostic criteria.

### 2.3 Florbetapir (F18-AV45) PET acquisition and preprocessing

The 18F-florbetapir (AV-45) tracer was employed to quantify amyloid burden [46]. The 18F-florbetapir PET scans were conducted using a Biograph mCT Flow PET/CT system (Siemens, Erlangen, Germany) 50 minutes after the intravenous administration of 7.4 MBq/kg (0.2 mCi/kg) of 18F-florbetapir, with each scan lasting 20 minutes. PET images were reconstructed using a filtered back-projection algorithm incorporating corrections for decay, and were realigned into a standardized image grid of 168 × 168 × 148 voxels, with each voxel measuring 2.04 × 2.04 × 1.5 mm. Corrections were also applied for normalization, dead time, photon attenuation, scatter, and random coincidences. All PET images were independently visually interpreted by three board-certified nuclear medicine physicians with expertise in the field, following established guidelines for visual rating, while remaining blinded to any other clinically relevant information [47]. PET images were defined by visual rating according to the guidelines for interpreting amyloid PET [47].

### 2.4 18F-MK-6240 PET and MRI

18F-MK6240 was prepared at the PET Center of Huashan Hospital, Fudan University. PET/CT imaging was conducted using a Siemens Biograph 64 PET/CT scanner (Siemens, Germany). Each subject received an intravenous injection of 18F-MK6240 at a dose of 5.55 MBq/kg, adjusted according to body weight. 20 minutes scans were conducted 90 minutes after intravenous injection of 18F-MK6240 [48]. Image reconstruction was completed using the filtered back projection algorithm (Filtered Back Projection, FBP), ensuring clear and reliable results. The 18F-MK6240 PET/CT brain imaging results for each participant were carefully reviewed by experienced attending two physicians. The standard uptake value (SUV) range was set between 0 and 3. Any areas showing abnormal uptake—either higher or lower—across two or more consecutive slices were marked for further attention. In cases where the two physicians had differing opinions on the uptake patterns within the Region of Interest (ROI), a third, more senior physician was invited to join the discussion to reach a conclusion.

Data were collected using a 3.0 Tesla scanner (SIEMENS MAGNE­TOM, Prisma 3.0 T, Siemens, Erlangen, Germany). Three-dimensional T1-weighted images were acquired using a magnetization-prepared rapid gradient-echo sequence in the sagittal plane with the following parameters: matrix = 320 × 320, field of view = 256 mm × 256 mm, slice thickness = 0.8mm, voxel size = 0.8mm × 0.8mm × 0.8mm, repetition time = 3000ms, echo time = 2.56ms, inversion time = 1100ms, flip angle = 7°, and number of slices = 208.

18F-MK-6240 PET image preprocessing was performed using the Statistical Parametric Mapping 12 (SPM12, https://www.fil.ion.ucl.ac.uk/spm/software/spm12). The 18F-MK6240 PET images are registered to the individual T1-weighted MRI images. Subsequently, the unified segmentation algorithm is employed to transform the T1 images into the standard space, and the transformation parameters are then applied to the corresponding 18F-MK6240 PET images. Based on the Braak stages and utilizing the Anatomical Automatic Labeling (AAL) atlas [49], gray matter brain templates for Braak stages 1 - 6 were constructed. Using the inferior portion of the cerebellum as the reference brain region, the standardized uptake value ratios (SUVR) of tau deposition across the respective Braak stages were calculated [50].

### 2.5 Measurements of plasma biomarkers

Testing was conducted on the Chemclin LiCA 800 automated immunoassay analyzer (Chemclin Diagnostics, Beijing, China) using the Chemclin LiCA® plasma Aβ42, Aβ40, p-tau181, p-tau217, NfL, and GFAP assay kits, in accordance with the manufacturer's instructions. EDTA-plasma samples were analyzed directly from aliquot tubes without prior cleanup, centrifugation, or dilution. The LiCA technology employs a dual-antibody sandwich immunoassay format involving two sequential incubation steps for the detection of blood-based biomarkers. In this system, one antibody is pre-conjugated to nano-scale Chemibeads, while the other is biotinylated. During the first incubation step, target analytes in the sample are simultaneously bound by both the Chemibead-immobilized detection antibody and the biotinylated capture antibody, forming a sandwich immune complex. In the second step, streptavidin-coated Sensibeads are introduced, which bind to the biotinylated antibody via the high-affinity biotin–streptavidin interaction. Upon laser excitation, the Sensibeads generate singlet oxygen molecules that diffuse to the adjacent Chemibeads, initiating a chemiluminescent signal. In the absence of the target analyte, no immune complex forms, and the beads remain spatially separated by more than 200 nm. This distance prevents energy transfer mediated by singlet oxygen, thereby minimizing nonspecific background signals. Notably, the LiCA assay operates entirely in a homogeneous phase, eliminating the need for washing steps commonly required in conventional immunoassays.

### 2.6 Statistical analyses

Demographics, scale scores, and biomarker data from all study participants were analyzed using SPSS (version 23.0; SPSS, Chicago, IL) and R 4.3.1 software (http://www.R-project.org). The Kolmogorov–Smirnov test was applied to assess the normality of variable distributions. For data with a normal distribution, a t-test was used to analyse the differences between groups. For data without a normal distribution, a Mann–Whitney test was performed. Continuous variables were summarized as mean ± standard deviation (SD). Categorical variables were presented as frequencies, and intergroup differences were analyzed using the Chi-square test or Fisher's exact test, as appropriate. The Benjamin/Hochberg (BH) method was employed to adjust p values for multiple comparisons in the analysis of demographics and scale scores [51]. Least absolute shrinkage and selection operator (LASSO) regression was implemented using the R "glmnet" package to address collinearity among independent variables and analyze high-dimensional data. In scenarios where the number of features exceeds the number of samples, traditional regression models often struggle with overfitting or poor generalization performance. However, Lasso's L1 regularization term helps imposing a penalty on the absolute values of the coefficients associated with each feature. Cross-validation was conducted with 10 folds, and the maximum number of iterations was set to 100. All variables were standardized before entering the LASSO regression and logistic regression. After selecting the variables in the LASSO, logistic regression analysis (“glm” function and R “rms” package) was used to further screen the variables and construct the model. Backward step-wise selection was applied in multiple logistic regression by using the likelihood ratio test with Akaike’s information criterion (AIC) as the stopping rule. Model performance was evaluated using the receiver operating characteristic (ROC) curve, with the “pROC” and “reportROC” packages utilized for visualization and assessment of the model's discriminatory ability. DeLong Test was used for comparison ROC curves. Partial correlation analysis was employed to examine the correlations between visuospatial memory and auditory verbal memory with both plasma biomarkers and tau pathology.

## 3 Results

### 3.1 Demographic and clinical characteristics

In this study, a total of 231 participants were analyzed, including 74 NC and 157 SCD, with 57.58 % females, and the mean age was 66.45 ± 7.59 years. Table 1 showed the demographic and neuropsychological features between the groups stratified by Aβ+ and Aβ- in individuals with NC and SCD. There was no significant difference between Aβ+ and Aβ- in NC group in age, education years, sex, and all neuropsychological assessments. In SCD group, there was no significant difference between subjects with Aβ+ and Aβ- in age, education years, and sex. The significant difference was found in the assessment of MoCA-B (P _unadjusted_ < 0.001, P _BH adjusted_ < 0.001), ACE-III (P _unadjusted_ < 0.05, P _BH adjusted_ < 0.05), AVLT-LD (P _unadjusted_ < 0.05), BVMT-LD (P _unadjusted_ < 0.001, P _BH adjusted_ < 0.001), BVMT-R (P _unadjusted_ < 0.01, P _BH adjusted_ < 0.05), BVMT-L (P _unadjusted_ < 0.001, P _BH adjusted_ < 0.001), BVMT-T (P _unadjusted_ < 0.001, P _BH adjusted_ < 0.001), STT-A (P _unadjusted_ < 0.05), STT-B (P _unadjusted_ < 0.05, P _BH adjusted_ < 0.05), DOC-SD (P _unadjusted_ < 0.001, P _BH adjusted_ < 0.05), and SMQ (P _unadjusted_ < 0.05). The difference of demographics and neuropsychological features between NC and SCD were showed in Supplementary Table 1.

**Table 1:** Difference of demographics and neuropsychological features between Aβ+ and Aβ- in participants with NC and SCD.

		NC		Aβ*+ vs* Aβ*-*	SCD		Aβ*+ vs* Aβ*-*	
		Aβ+ (*n* = 7)	Aβ- (*n* = 67)	*t/P*	Aβ+ (*n* = 31)	Aβ- (*n* = 126)	*t(χ²)/P*	*P_BH adjusted_*
	age	62.29, 9.88	65.40, 7.49	−1.01/0.314	69.29, 5.75	66.75, 7.25	−1.81/0.072	0.144
	sex (M/F)	4/3	22/45	0.232*	19/12	53/73	3.70/0.054	0.117
	education	14.43, 1.90	12.18, 3.88	1.51/0.136	11.94, 2.68	12.71, 2.96	1.34/0.184	0.319
Global cognition	MoCA-B ACE-III	25.00, 2.58	25.98, 2.34	−1.05/0.299	22.19, 3.58	24.90, 3.00	4.34/0.000	<0.001
		82.43, 8.02	81.89, 7.10	0.19/0.852	76.39, 8.39	80.56, 8.17	2.53/0.012	<0.05
Memory	AVLT-LD	6.43, 1.40	6.22, 1.89	0.29/0.773	3.32, 2.73	4.47, 2.57	2.20/0.029	0.075
	AVLT-R	22.43, 0.98	22.31, 1.32	0.23/0.815	21.16, 1.71	21.40, 2.22	0.55/0.582	0.796
	BVMT-LD	9.29, 2.21	9.34, 2.50	−0.05/0.957	5.52, 3.35	9.17, 2.73	6.37/0.000	<0.001
	BVMT-R	11.86, 0.38	11.68, 0.83	0.57/0.574	10.61, 1.75	11.52, 1.05	2.79/0.009	<0.05
	BVMT-L	5.86, 2.19	5.57, 2.43	0.30/0.765	3.21, 1.97	5.44, 2.59	4.48/0.000	<0.001
	BVMT-F	2.14, 2.10	2.09, 2.27	0.55/0.957	4.87, 2.45	2.39, 2,27	−5.27/0.000	<0.001
Executive	STT-A	53.14, 21.32	46.38, 19.43	0.87/0.389	57.65, 22.52	48.76, 14.68	−2.69/0.044	0.104
	STT-B	135.88, 27.50	133.74, 41.73	0.13/0.896	161.23, 56.29	134.14, 36.98	−3.26/0.015	<0.05
Language	AFT	19.43, 7.35	18.18, 4.63	0.64/0.527	16.23, 5.24	17.37, 4.85	1.15/0.250	0.406
	BNT	24.86, 3.02	24.82, 2.72	0.04/0.970	23.77, 3.55	24.11, 3.90	0.45/0.657	0.854
visuo-spatial	JLO	17.17, 3.54	20.09, 4.67	−1.49/0.141	20.50, 4.98	20.90, 4.69	0.40/0.687	0.851
Attention	DST	12.29, 1.98	12.88, 2.27	−0.66/0.510	12.23, 1.81	12.59, 2.52	0.72/0.470	0.719
	DSTb	5.00, 0.82	5.03, 1.49	−0.05/0.957	4.40, 1.07	4.80, 1.54	1.35/0.180	0.334
	DSTf	7.29, 1.60	7.85, 1.19	−1.15/0.256	7.83, 1.34	7.87, 1.34	0.12/0.907	1.000
Metacognition	DOC-SD	1.08, 0.23	1.06, 0.16	0.20/0.845	1.22, 0.17	1.11, 0.15	−3.59/0.000	<0.05
	DOC-LD	0.88, 0.14	0.95, 0.16	−1.25/0.217	1.04, 0.25	1.06, 0.15	0.23/0.817	0.966
	SMQ	3.00, 2.53	4.60, 3.17	−1.20/0.234	7.54, 3.63	6.06, 3.13	−2.30/0.023	0.066
Mood	HAMD	3.17, 3.19	5.80, 6.58	−0.57/0.339	5.71, 4.23	6.73, 7.26	0.08/0.938	1.000
	HAMA	2.83, 3.71	3.63, 3.24	−0.96/0.572	5.18, 4.29	5.10, 5.22	−0.71/0.480	0.693

MoCA-B, Chinese version of Montreal Cognitive Assessment-Basic; ACE-III, Chinese version of Addenbrooke’s Cognitive Examination III; AVLT, Auditory Verbal Learning Test; LD, long-term delayed recall; R, recognition; Brief Visuospatial Memory Test, BVMT; L, learning; F, forgetting; STT-A, STT-B, Shape Trail Test Part A and B; AFT, Animal Verbal Fluency Test; BNT, Boston Naming Test; JLO, Judgement of Line Orientation; DST, Digit Span Test; DSTb, Digit Span Test-backwards; DSTf, Digit Span Test-forwards; DOC, Degree of Confidence; DOC-SD, Degree of Confidence-short-term delayed recall; DOC-LD, Degree of Confidence-long-term delayed recall; SMQ, self-report memory questionnaire; HAMD, Hamilton Depression Rating Scale; HAMA, Hamilton Anxiety Rating Scale; NC, normal controls; SCD, subjective cognitive decline. The score range: MoCA‑B, 0-30; ACE-III, 0-100; AVLT-LD, 0-12; AVLT-R, 0-24; BVMT-LD, 0-12; BVMT-R, 0-12; BVMT-L, 0-12; BVMT-F, 0-12; STT-A, STT-B, AFT have no maximum score; BNT, 0-30; JLO, 0-30; DST, 0-22; DSTb,0-10, DSTf, 0-12; DOC-SD, 0-2; DOC-LD, 0-2; SMQ, 0-18; HAMD, 0-52, HAMA, 0-56. *: Fisher's exact test. Data are presented as mean, SD. The last two columns list the unadjusted and adjusted p values according to Benjamini/Hochberg (BH).

The difference of tau PET SUVr and plasma biomarkers between Aβ+ and Aβ- in participants with NC and SCD were listed in Table 2. No significant differences were observed in tau PET SUVr (Braak I-II, Braak III-IV, and Braak V-VI), p-tau217, p-tau181, NFL, GFAP, or Aβ42/Aβ40 levels between Aβ+ and Aβ- participants in the NC group. In the SCD group, tau PET SUVr values in Braak I-II, Braak III-IV, and Braak V-VI were significantly higher in Aβ+ participants compared to Aβ− participants (*p* < 0.001, *p* < 0.001, and *p* < 0.05, respectively). Additionally, levels of p-tau217, p-tau181, and GFAP were significantly elevated in the Aβ+ group compared to the Aβ− group (all *p* < 0.001). The Aβ42/Aβ40 ratio was significantly lower in the Aβ+ group than in the Aβ− group (*p* < 0.005).

**Table 2:** Difference of tau PET SUVr and plasma biomarkers between Aβ+ and Aβ- in participants with NC and SCD.

	NC		Aβ*+ vs* Aβ*-*	SCD		Aβ*+ vs* Aβ*-*
	Aβ+ (*n* = 7)	Aβ- (*n* = 67)	*t/P*	Aβ+ (*n* = 31)	Aβ- (*n* = 126)	*t/P*
Braak I-II	0.86, 0.09	0.85, 0.11	0.31/0.759	1.10, 0.30	0.85, 0.10	−7.21/0.000
Braak III-IV	0.91, 0.07	0.91, 0.09	−0.11/0.913	1.01, 0.13	0.91, 0.09	−3.68/0.001
Braak V-VI	0.91, 0.08	0.93, 0.09	−0.53/0.602	0.97, 0.10	0.92, 0.08	−2.52/0.013
Braak-like visual stage (I/II/III/IV)*	7/0/0/0	–	–	12/7/9/0	–	–
p-tau217	0.35, 0.16	0.34, 0.12	0.14/0.892	0.76, 0.43	0.32, 0.13	−9.98/0.000
p-tau181	4.79, 0.71	4.67, 0.89	0.33/0.747	5.38, 0.83	4.53, 0.86	−4.96/0.000
NfL	25.79, 7.32	27.45, 7.50	−0.55/0.583	30.60, 8.12	28.64, 9.66	0.27/0.301
GFAP	119.80, 53.01	117.32, 41.50	0.15/0.885	202.17, 101.17	126.26, 50.40	−4.06/0.000
Aβ42/Aβ40	0.03, 0.01	0.03, 0.01	−1.21/0.230	0.02, 0.00	0.04, 0.02	2.86/0.005

Unit of plasma biomarkers: pg/ml. *: Tau vision results(I/II/III/IV), I means A+T_2-_, II means A+T_2MTL+_, III means A+T_2MOD+_, IV means A+T_2HIGH+_, 3 subjects in stage 2 with A+T _Braak_^atypical+^.

### 3.2 Establishment of Three Types of Predictive Models

Three types of predictive models were established for distinct purposes (Table 3). Model 1 is a neuropsychology-based model designed to identify the most sensitive cognitive markers among a comprehensive neuropsychological battery. To achieve robust feature selection and dimensionality reduction, LASSO regression was applied to eight candidate variables—MoCA-B, ACE-III, BVMT-LD, BVMT-R, BVMT-F, BVMT-T, STT-B, and DOC-SD—prior to constructing the final predictive model. BVMT-LD and MoCA-B were identified as the most optimal predictors following LASSO regression filtration (Supplementary Fig. 1). Then, logistic regression (LR) analysis was conducted with BVMT-LD and MoCA-B included as independent variables. Results were reported as odds ratios (95 % CI): BVMT-LD (0.37 [0.23, 0.61]) and MoCA-B (0.88 [0.42, 1.06]). Model 2 focused on validating the predictive value of established plasma biomarkers. Given the relatively small number of plasma variables, LR was employed directly to identify the optimal predictive combination. In the univariable LR, the plasma levels of p-tau217, p-tau181, GFAP, and the Aβ42/Aβ40 were significantly associated with Aβ+ status in the SCD group (all with *p* < 0.05). (see Supplementary Table 2). Those variables were further included in multivariable LR using backward selection, p-tau217 and Aβ42/Aβ40 were retained in the final model, with corresponding odds ratios (95 % CI) of 7.50 (2.52, 22.33) and 0.04 (0, 0.46), respectively.

**Table 3:** Logistic regression analysis of neuropsychology-based (Model 1), plasma-based (Model 2), and multimodal feature-based (Model 3) models for predicting amyloid-β in individuals with SCD.

Model	Variables	β	OR (95 % CI)	P value	AIC
Model 1	BVMT-LD	−0.99	0.37 (0.23, 0.61)	0.000	128.13
	MoCA-B	−0.41	0.88 (0.42, 1.06)	0.089	
Model 2	p-tau217	2.01	7.50 (2.52, 22.33)	0.000	88.41
	Aβ42/Aβ40	−3.18	0.04 (0, 0.46)	0.009	
Model 3	BVMT-LD	−0.96	0.38 (0.21, 0.69)	0.001	84.96
	p-tau217	2.46	11.66 (4.04, 33.62)	0.000	

Backward step-wise selection was applied, by using the likelihood ratio test with Akaike’s information criterion (AIC) as the stopping rule.

**Fig. 2:**
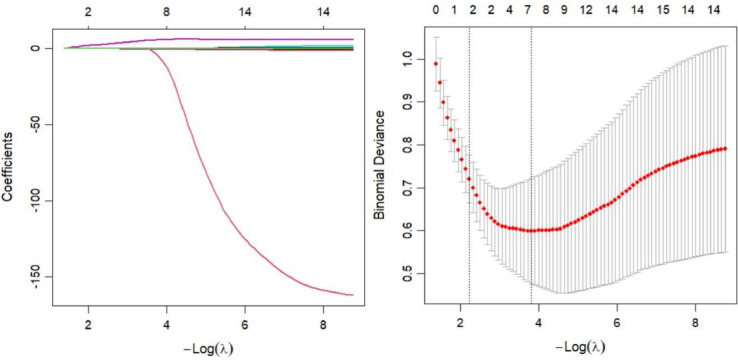
Features selection by least absolute shrinkage and selection operator (LASSO).

Model 3 is a multimodal variables model that aims to incorporate demographic, neuropsychological, and plasma variables to establish a predictive model. Features were selected using LASSO regression for simultaneous dimensionality reduction and feature selection. The initial set of 15 candidate variables, including age, sex, education, MoCA-B, ACE-III, BVMT-LD, BVMT-R, BVMT-F, BVMT-T, STT-B, DOC-SD, p-tau217, p-tau181, GFAP, and Aβ42/Aβ40, was reduced to a final model containing two features: BVMT-LD and p-tau217. (Fig.2). Then, BVMT-LD and p-tau217 were included in logistic regression analysis, with corresponding odds ratios (95 % CI) of 0.38 (0.21, 0.69) and 11.66 (4.04, 33.62), respectively.

(Left) A coefficient profile plot was produced against the log lambda sequence. LASSO coefficients profiles (y-axis) of the 15 features, including age, sex, education, MoCA-B, ACE-III, BVMT-LD, BVMT-R, BVMT-F, BVMT-T, STT-B, DOC-SD, p-tau217, p-tau181, GFAP, Aβ42/Aβ40. (Right) Ten-fold cross-validation for tuning parameter selection in the LASSO model. The partial likelihood deviance (binomial deviance) curve was plotted versus log(λ). To avoid overfitting, 1 standard error was selected.

### 3.3 Diagnostic performance of different Models

**Fig. 3:**
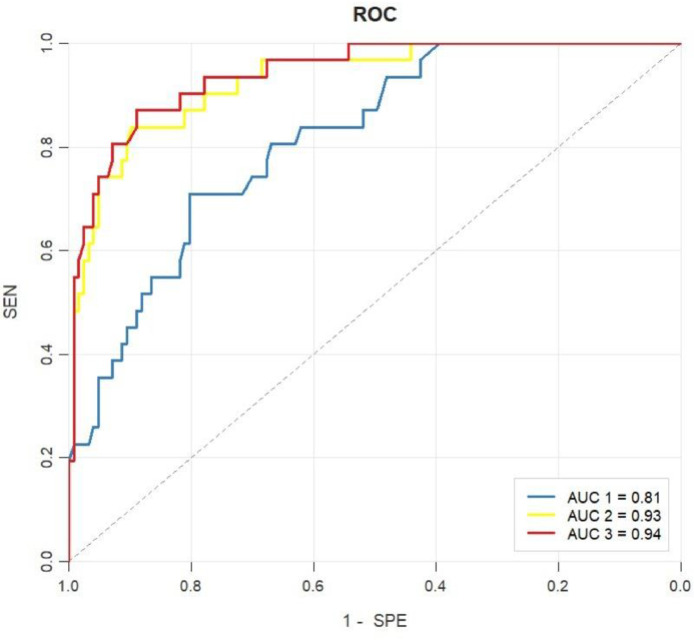
ROC curves of the models for prediction Aβ+ or Aβ- in SCD.

The analysis results of the ROC curves were displayed (Fig.3). The diagnostic performance of different models is presented in Table 4. The model 1 yielded an AUC of 0.82 to identify Aβ+ participants from Aβ-, with the ACC, SEN, SPE, PLR, NLR, PPV, and NPV of 0.79, 0.71, 0.80, 3.61, 0.36, 0.47 and 0.92, respectively. The model 2 yielded an AUC of 0.93, with the ACC, SEN, SPE, PLR, NLR, PPV, and NPV of 0.89, 0.84, 0.90, 8.19, 0.18, 0.67 and 0.96, respectively. The model 3 yielded an AUC of 0.94, with the ACC, SEN, SPE, PLR, NLR, PPV, and NPV of 0.89, 0.87, 0.89, 7.90, 0.15, 0.66 and 0.97, respectively. In addition, DeLong test was used to compare the areas under two ROC curves. The AUC of Model 1 value was significantly lower than that of both Model 2 and Model 3 (0.82 vs. 0.93, *Z* = −2.82; 0.82 vs. 0.94, *Z* = −4.02), with all comparisons yielding *p* < 0.05. The AUC value of Model 3 was marginally higher than that of Model 2 (0.94 vs. 0.93, *Z* = −0.76); however, this difference was not statistically significant (*p* > 0.05).

The three curves illustrate the performance of the three predictive models. Blue line: Model 1 (neuropsychology-based). This model incorporates BVMT-LD and MoCA-B as independent variables and achieves an AUC 1 of 0.81. Yellow line: Model 2 (plasma-based). This model retains p-tau217 and the Aβ42/Aβ40 as predictors and yields an AUC 2 of 0.93. Red line: Model 3 (multimodal feature-based). This model combines BVMT-LD and p-tau217 as predictors and attains an AUC 3 of 0.94.

**Table 4:** Accuracy of the prediction of the models.

Model	AUC	Accuracy (ACC)	Sensitivity (SEN)	Specificity (SPE)	Positive likelihood ratio (PLR)	Negative likelihood ratio (NLR)	Positive predictive value (PPV)	Negative predictive value (NPV)
Model 1	0.82	0.79 (0.78, 0.79)	0.71 (0.55, 0.87)	0.80 (0.73, 0.87)	3.61 (2.38, 5.47)	0.36 (0.21, 0.63)	0.47 (0.33, 0.61)	0.92 (0.87, 0.97)
Model 2*	0.93	0.89 (0.89, 0.89)	0.84 (0.71, 0.97)	0.90 (0.85, 0.95)	8.19 (4.79, 14.03)	0.18 (0.08, 0.40)	0.67 (0.52, 0.82)	0.96 (0.92, 1.00)
Model 3^#, &^	0.94	0.89 (0.89, 0.89)	0.87 (0.75, 0.99)	0.89 (0.84, 0.94)	7.90 (4.73, 13.19)	0.15 (0.06, 0.36)	0.66 (0.51, 0.80)	0.97 (0.93, 1.00)

DeLong test was used to compare the areas under two ROC curves. *: AUC1 vs AUC2, *Z* = −2.82, *p* = 0.004. #: AUC1 vs AUC3, *Z* = −4.02, *p* = 0.000. &: AUC2 vs AUC3, *Z* = −0.76, *p* = 0.445. PPV and NPV were calculated based on the observed prevalence of Aβ positivity in the SCD cohort (31/157, 19.7 %).

### 3.4 Partial correlation between visuospatial verbal learning test, auditory verbal memory test, and biomarkers

Table 5 presents the partial correlation analysis between visual and auditory verbal learning tests and biomarkers in SCD with Aβ+ status, adjusted for age. The AVLT-LD scores showed a significant negative correlation with the tau vision stage and p-tau217 levels (*p* < 0.01 and *p* < 0.05, respectively). The AVLT-R scores demonstrated a significant negative correlation with the tau vision stage (*p* < 0.05). The BVMT-LD showed a significant negative correlation with p-tau217 and GFAP levels (all *p* < 0.05). Notably, GFAP exhibited a strong negative correlation with BVMT-LD (*r* = −0.454, *p* < 0.05), which was one of the most robust correlations observed between plasma biomarkers and cognitive measures. This suggests that astrocyte activation (reflected by elevated GFAP) may be closely linked to visuospatial memory impairment in SCD individuals with Aβ+, potentially indicating that neuroinflammation contributes to early cognitive decline in preclinical AD.

**Table 5:** Partial correlation between auditory verbal learning test, visuospatial memory test, and biomarkers in SCD with Aβ+.

	AVLT-LD	AVLT-R	BVMT-LD	BVMT-R	BVMT-F	BVMT-T
Tau stage (vision)	−0.512**	−0.465*	−0.23	−0.141	−0.207	−0.207
p-tau217	−0.422*	−0.312	−0.386*	−0.371	−0.27	−0.331
p-tau181	−0.229	−0.179	−0.1	−0.286	0.049	−0.015
Aβ42/Aβ40	0.247	0.292	0.235	−0.102	0.279	0.174
GFAP	−0.213	−0.253	−0.454*	−0.338	−0.029	−0.328
NFL	−0.215	0.191	−0.326	−0.339	−0.016	−0.296

Age was adjusted in all analysis. *: *p* < 0.05; **: *p* < 0.01.

## 4 Discussion

In this study, SCD individuals with Aβ+ showed worse global cognition, visuospatial memory, executive function, and metamemory, higher tau PET SUVr, elevated p-tau217, p-tau181, and GFAP levels, and lower Aβ42/Aβ40 ratios than SCD individuals with Aβ- individuals. No significant differences in demographics, cognitive assessments, plasma biomarkers, or tau PET SUVr were found between Aβ+ and Aβ- individuals in the NC group. Subsequently, we established three types of models to predict Aβ status in SCD. Both Model 2 and Model 3 demonstrated high predictive performance, achieving AUC values of 0.93 and 0.94, respectively, probably representing the highest reported levels to date among prediction models specifically developed for the SCD population. Lastly, a partial correlation analysis was conducted in SCD with Aβ+. The results revealed that both auditory verbal memory (AVLT-LD) and visuospatial memory (BVMT-LD) were significantly negatively correlated with p-tau217, whereas only AVLT-LD showed a significant negative association with Braak-like visual stage. These findings indicate that, in this population, auditory memory performance is more strongly linked to the severity of tau pathology, yet less sensitive than visuospatial memory in detecting amyloid deposition. Notably, Model 2 (p-tau217 + Aβ42/Aβ40) achieved an AUC of 0.93, which was marginally lower than Model 3 (BVMT-LD + p-tau217, AUC=0.94) but not statistically different (*p* = 0.445). This finding highlighted that a blood-only biomarker panel can serve as a highly effective tool for identifying preclinical AD in SCD individuals. The comparable performance between the two models underscores the dual value of plasma biomarkers and cognitive measures in early AD detection, providing flexible options for different clinical scenarios.

Due to the small sample size of the NC Aβ+ group (*n* = 7), no statistically significant differences were detected between NC Aβ+ and NC Aβ- groups in cognitive assessments, plasma biomarkers, or tau PET SUVr. However, these results should be interpreted with caution, as the limited sample size may have reduced the ability to detect potential subtle differences. Replication in larger, well-characterized cohorts is therefore warranted to validate these results. Subtle deficits in global cognition (MoCA-B, ACE-III), visuospatial memory (AVLT-LD, BVMT-LD, R, L, and F), executive function (STT-A, STT-B), and metacognition (DOC-SD, SMQ) are detectable in Aβ+ SCD individuals, although these minor impairments are not sufficient for a clinical diagnosis of MCI and occur concurrently with tau pathology and abnormal blood biomarkers. Aβ+ SCD individuals had significantly higher tau-PET SUVr (Braak I–II, Braak III–IV, and Braak V–VI), p-tau217, p-tau181, and GFAP levels compared to Aβ- individuals, and significantly lower Aβ42/Aβ40 ratios compared to Aβ- individuals in SCD group.

In our study, we observed that SCD with Aβ deposition (stage 2), auditory memory appears to be more susceptible to impairment compared to visual memory. The association between the hippocampus and episodic memory is well established. However, compared to verbal episodic memory, visuospatial episodic memory has been less frequently examined in preclinical AD research. One longitudinal study indicated that a decline in visual memory, as measured by paired associates learning (PAL) and delayed matching to sample (DMS) tests, could effectively distinguish between different types of MCI [52]. Another study found a positive association between BVMT immediate recall scores and hippocampal subfields in individuals with MCI[53]. In comparison with the verbal memory test (AVLT-H), the visuospatial memory test (BVMT) requires participants to simultaneously remember both the shapes and positions of the images, which may make the task more complex. The abstract and unfamiliar graphics used in the BVMT may demand greater learning efforts compared to simple storage functions. In addition, the superior sensitivity of the BVMT may reflect the involvement of a broader network of cerebral regions rather than merely a more demanding task. Visuospatial memory engages both the dorsal (“where/how”) and ventral (“what”) visual processing streams [54]. The dorsal pathway mediates spatial positioning and motion processing, and the ventral pathway subserves object recognition; their integration—termed multi-regional cerebral connectivity—is uniquely vulnerable in early AD [54], which likely accounts for the heightened discriminative power of the BVMT compared with the purely verbal AVLT.

In the next step, LASSO regression was used to further screening the variables [55,56], which is a regularization technique that has proven effective for selecting the most relevant features for the response variable. The initial set of 15 features was reduced to a final model containing two features, BVMT-LD (long-term delayed recall) and p-tau217. Then, we compared three logistic regression models with different variable combinations, and Model 3, which incorporated BVMT-LD and p-tau217, demonstrated the highest AUC value in differentiating Aβ status in individuals with SCD, although no statistically significant difference was observed between Model 3 and Model 2. Model 2 is a plasma-based predictive model incorporating p-tau217 and Aβ42/Aβ40, while Model 3 is a multimodal feature-based model that includes a neuropsychological variable (BVMT-LD) and a plasma biomarker (p-tau217). Model 3 demonstrated performance comparable to that of the reference model based on key plasma biomarkers p-tau217 and Aβ42/Aβ40, with no statistically significant difference observed. This indicates that BVMT-LD plays a crucial role in the predictive model and underscores the potential value of neuropsychological markers at the SCD stage. Compared with the combined detection of multiple plasma biomarkers or the gold standard of Aβ PET imaging, the use of this dual-index panel reduces the overall diagnostic cost while maintaining high diagnostic accuracy, which is particularly suitable for large-scale screening in primary medical institutions with limited resources. In addition, Aβ42/Aβ40 detection is significantly more demanding than p-tau217 due to inherent molecular properties and analytical requirements. Plasma Aβ42 and Aβ40 are highly susceptible to aggregation (forming oligomers or fibrils) and proteolytic degradation, necessitating stringent pre-analytical quality control [57]. Even the tube material, tube size, the presence of gel separators, and the freeze–thaw cycles were found to influence the quantification of plasma Aβ40 and Aβ42 [[58], [59], [60], [61]]. At room temperature (RT), Aβ levels start to decline within 1 hour of phlebotomy (prior to centrifugation): reductions reach up to 5 % at 2 hours and 8–10 % by 6 hours [57], with consistent findings showing 5 % and 10 % decreases at these respective time points [58]. For separated plasma, delayed measurement further impacts Aβ concentrations, with reductions of up to 7 % after 6 hours and 10 % after 24 hours of storage [58]. Notably, regardless of the detection assay used, a 24-hour delay in centrifugation at RT leads to substantial declines in plasma Aβ42 (59–81 %) and Aβ40 (62–93 %), accompanied by a reduction in the Aβ42/Aβ40 ratio (59–85 %) [60]. In contrast, refrigeration at 4 °C effectively preserves Aβ integrity, although the Aβ42/Aβ40 ratio enhances diagnostic specificity, it fails to fully mitigate pre-analytical variability [60]. By comparison, phosphorylated tau (p-tau) isoforms, NfL, and GFAP exhibit markedly greater pre-analytical robustness. For instance, p-tau isoforms, NfL, and GFAP concentrations remain unchanged when stored at RT for 24 hours [57,61], making them more resilient to real-world pre-analytical fluctuations. In resource-limited primary care settings or regions with underdeveloped laboratory infrastructure, the implementation of standardized pre-analytical workflows for Aβ42/Aβ40 may be impractical or lacking entirely. Even in settings where p-tau217 can be reliably measured, Aβ42/Aβ40 detection may remain unfeasible due to these technical barriers. In contrast, BVMT-LD is a cost-effective, easy-to-administer neuropsychological test that requires no specialized equipment—only standard test materials and trained clinicians. By integrating BVMT-LD with p-tau217, Model 3 provides a "flexible alternative" that circumvents the need for Aβ42/Aβ40 detection while maintaining high diagnostic accuracy (AUC=0.94). Notably, GFAP showed a significant negative correlation with BVMT-LD (*r* = −0.454, *p* < 0.05) in SCD Aβ+ individuals, suggesting that visuospatial memory impairment may be linked to early neuroinflammation, which is consistent with the role of GFAP in reflecting AD-related glial activation

Plasma p-tau217 is an AD neuropathology biomarker. Elevated plasma p-tau217 is detectable in asymptomatic early stages, reflecting abnormal tau phosphorylation that coincides with Aβ deposition and indicates a critical therapeutic window [62]. Moreover, blood p-tau217 has demonstrated potential in predicting the progression in AD [62]. Palmqvist et al. (2020) found that in 1,402 participants from three cohorts, plasma p-tau217 had an AUC of 0.90–0.93 (sensitivity and specificity >90 %), comparable to CSF testing and PET imaging, and could independently diagnose AD [62]. Blood p-tau217 higher accuracy in differentiating AD from other neurodegenerative diseases, with an AUC value higher than that of p-tau181, NFL, and MRI [62]. According to recent multiple large-scale independent studies, in individuals with MCI or AD dementia, plasma p-tau217 demonstrates the ability in differentiating Aβ positive from negative individuals with an AUC ranging from 0.90 to 0.96 [10,[63], [64], [65]]. Plasma p-tau217 is a highly powerful blood biomarker for predicting Aβ status in the overall AD population. However, studies specifically focusing on SCD individuals remain limited—SCD is often investigated alongside MCI or pre-dementia AD stages—and current evidence indicates its predictive accuracy in SCD is comparable to, or only slightly lower than, that in MCI, especially when combined with other biomarkers [66]. In our study, both Model 2 and Model 3 demonstrated high predictive performance, achieving AUC values of 0.93 and 0.94, respectively. To date, most predictive models have been derived from heterogeneous samples that include individuals with MCI or Alzheimer’s disease dementia [10,[63], [64], [65]], rather than from a pure SCD cohort. Our findings may represent the highest reported levels to date among prediction models specifically developed for the SCD population.

Episodic memory impairment is the core clinical feature of patients with typical AD at an early stage; the initial clinical presentation is typically characterized by short-term memory deficits, followed by progressive impairment in other cognitive domains [67]. BVMT assesses visuospatial episodic memory, a subtype of episodic memory. Although research on visuospatial episodic memory tests in SCD remains relatively limited, due to its less widespread use in clinical practice compared to auditory verbal memory assessments. Recent years, emerging evidence indicated that visuospatial episodic memory demonstrate high sensitivity to early-stage cognitive impairment [53,68]. In our study, the *Post-hoc* subgroup analysis confirmed that BVMT-LD exhibited a more significant effect in distinguishing SCD Aβ+ from Aβ- individuals (*p* < 0.001, BH-adjusted) compared with AVLT-LD (*p* = 0.029, unadjusted; *p* = 0.075, BH-adjusted). During the LASSO regression analysis for Model 1 (cognitive-based model) and Model 3 (multimodal model), BVMT-LD showed a stronger correlation with Aβ PET positivity than AVLT. We further to examine whether the two types of memory test yield distinct correlations with biomarkers. The results revealed that AVLT indices were primarily correlated with tau pathology (significantly negative correlations with plasma p-tau217 and tau PET visual staging, *p* < 0.05). In contrast, BVMT-LD showed dual correlations: it was not only negatively associated with plasma p-tau217 but also significantly correlated with GFAP —a marker reflecting astrocyte activation downstream of tau pathology. Given that our predictive models were constructed to identify Aβ PET+, BVMT-LD’s linkage to both tau and downstream neuroinflammatory processes likely enhanced its predictive value for Aβ-related pathological change.

### 4.1 Limitation

This was a cross-sectional study, so we cannot establish causal relationships between cognitive deficits, plasma biomarkers, and amyloid/tau pathology. Longitudinal follow-up is needed to validate the predictive value of BVMT-LD and p-tau217 for disease progression. The small sample size of the NC Aβ+ subgroup limits statistical power; larger, multicenter NC Aβ+ cohorts are required to validate the current findings. The cohort was restricted to the populations in eastern China, limiting generalizability. We propose expanding the recruitment scope of the Chinese Preclinical Alzheimer’s Disease Study (C-PAS) to include geographically diverse populations in future phases, to enhance the external validity of the study results.

## 5 Conclusion

In this study, we found that visuospatial memory (BVMT-LD) may serve as potential cognitive marker in the SCD stage. We evaluated a predictive model combining the BVMT-LD with p-tau217 to identify amyloid pathology, which demonstrated an AUC of 0.94. This model was selected through LASSO regression analysis as the most optimal predictive combination, although no statistically significant difference of AUC values was observed between Model with BVMT-LD+ p-tau217 and Model with p-tau217 + Aβ42/Aβ40. Lastly, we found that, in SCD with Aβ+, auditory memory performance was more closely associated with the severity of tau pathology, but less sensitive than visuospatial memory in detecting amyloid deposition. According to the latest AA criteria established in 2024, stages 1 to 2 constitute the optimal therapeutic window for targeted disease-modifying therapies (DMTs), such as anti-amyloid-β (anti-Aβ) agents. Initiating pharmacological intervention during these stages may halt the pathological progression prior to the formation of tau protein tangles and neuronal loss, thereby preventing or delaying the onset of clinical symptoms. Our study highlights the potential of visuospatial memory performance and plasma p-tau217 as non-invasive biomarkers for assessing amyloid-β pathology in the preclinical stage of Alzheimer's disease.

## Funding

This work was supported by The 10.13039/501100001809National Natural Science Foundation of China (82171198); Shanghai Municipal Science Technology Major Project (No. 2018SHZDZX01); STI2030-Major Projects (2022ZD0213800).

## Declaration of generative AI and AI-Assisted technologies in the writing process

We confirm that we have not used any AI and AI-Assisted technologies in the writing process.

## Data availability statement

The data that support the findings of this study are available on request from the corresponding author.

## CRediT authorship contribution statement

**Qinjie Li:** Writing – review & editing, Writing – original draft, Formal analysis, Conceptualization. **Lin Huang:** Data curation. **Ying Wang:** Investigation. **Yihui Guan:** Resources. **Fang Xie:** Software. **Qihao Guo:** Supervision, Funding acquisition.

## Declaration of competing interest

The authors declare that they have no conflicts of interest.

## Appendix Supplementary materials

Image, application 1
