# Supplementary material for: Visuospatial memory deficit, plasma p-tau217, and Aβ42/Aβ40 ratio enhance sensitivity to identify Aβ PET positivity in individuals with SCD
Source: J Prev Alzheimers Dis. 2026 Feb 27;13(5):100525. doi: 10.1016/j.tjpad.2026.100525 (PMC12964029; doi:10.1016/j.tjpad.2026.100525)
Supplement: Supplementary file 1 [file mmc1.docx]

Supplementary Table 1. Difference of demographics and neuropsychological features between NC and SCD.

|  |  | | NC (n=74) | SCD (n=157) | P |
| --- | --- | --- | --- | --- | --- |
|  | | age | 64.94, 7.66 | 66.99, 7.47 | 0.059 |
|  | | sex (M/F) | 26/48 | 72/85 | 0.124 |
|  | | education | 12.37, 3.80 | 12.57, 3.15 | 0.682 |
| Global cognition | | MoCA-B  ACE-III | 25.89, 2.38 | 24.44, 5.25 | <0.001 |
|  |  |  | 81.67, 7.04 | 79.67, 9.72 | 0.063 |
| Memory | | AVLT-LD | 6.23, 1.85 | 4.31, 2.66 | <0.001 |
|  | | AVLT-R | 22.31, 1.29 | 21.37, 2.11 | <0.001 |
|  | | BVMT-LD | 9.28, 2.43 | 8.47, 3.30 | <0.05 |
|  | | BVMT-R | 11.69, 0.80 | 11.29, 1.46 | <0.01 |
|  | | BVMT-L | 5.58, 2.41 | 4.95, 2.65 | 0.077 |
|  | | BVMT-F | 2.10. 2.23 | 2.78, 2.35 | <0.05 |
| Executive | | STT-A | 46.96, 19.70 | 50.60, 16.93 | 0.148 |
|  |  | STT-B | 134.21, 40.65 | 139.69, 44.07 | 0.463 |
| Language | | AFT | 18.27, 4.93 | 17.19, 4.80 | 0.117 |
|  |  | BNT | 24.79, 2.73 | 24.05, 3.73 | 0.136 |
| visuo-spatial | | JLO | 19.80, 4.66 | 20.83, 4.70 | 0.125 |
| Attention | | DST | 12.79, 2.24 | 12.54, 2.40 | 0.446 |
|  |  | DSTb | 5.01, 1.44 | 4.75, 1.47 | 0.198 |
|  |  | DSTf | 7.77, 1.23 | 7.85, 1.34 | 0.695 |
| Metacognition | | DOC-SD | 1.07, 0.17 | 1.13, 0.16 | <0.01 |
|  |  | DOC-LD | 0.94, 0.15 | 1.05, 0.21 | <0.001 |
|  |  | SMQ | 4.46, 3.14 | 6.33, 3.32 | <0.001 |
| Mood | | HAMD | 5.48, 6.50 | 6.53, 6.93 | <0.01 |
|  |  | HAMA | 3.50, 3.31 | 5.15, 5.10 | 0.365 |

Supplementary Table 2. Univariable logistic regression analysis of plasma biomarkers in individuals with SCD stratified by Aβ status

| Variables | β | OR (95% CI) | P value |
| --- | --- | --- | --- |
| p-tau 217 | 2.75 | 15.67 (5.37, 45.76) | 0.000 |
| p-tau 181 | 1.46 | 44.30 (2.20, 8.42) | 0.000 |
| NfL | 0.19 | 1.22 (0.84, 1.76) | 0.303 |
| GFAP | 1.04 | 2.83 (1.73, 4.62) | 0.000 |
| Aβ42/Aβ40 | 1.14 | 0.00 (0.00, 0.23) | 0.000 |


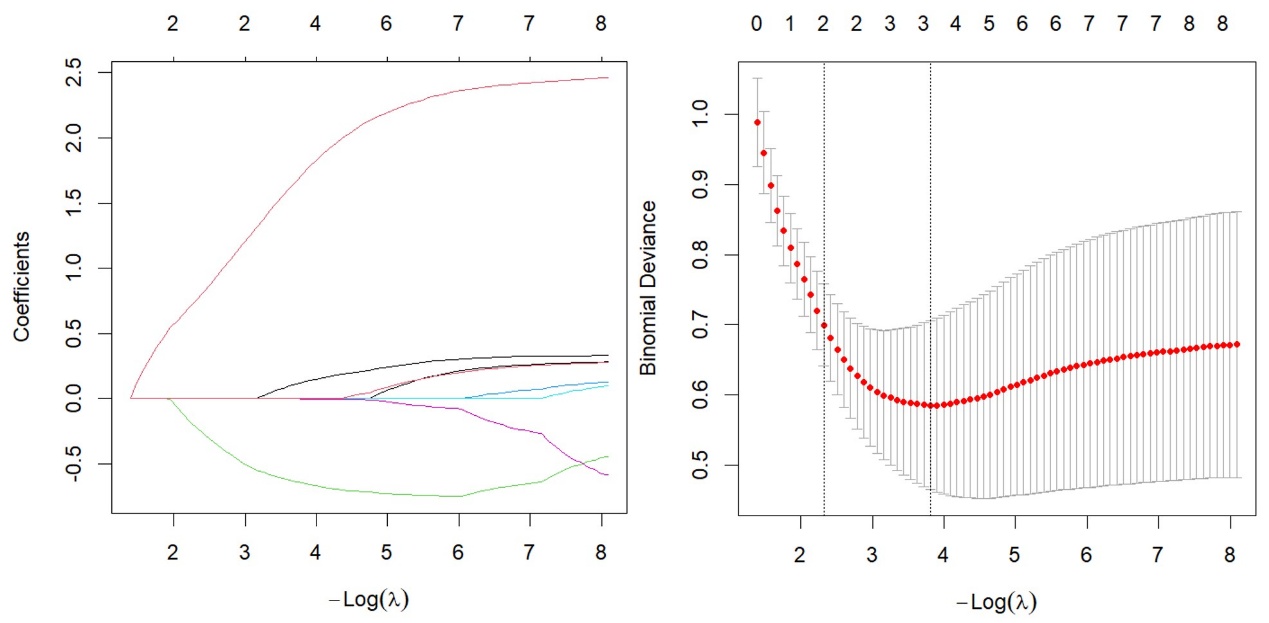


Supplementary Fig. 1 Features selection by least absolute shrinkage and selection operator (LASSO) in eight neuropsychological variables
